# Comparison of the antimicrobial consumption in weaning pigs in Danish sow herds with different vaccine purchase patterns during 2013

**DOI:** 10.1186/s40813-016-0042-1

**Published:** 2016-10-01

**Authors:** Carolina Temtem, Amanda Brinch Kruse, Liza Rosenbaum Nielsen, Ken Steen Pedersen, Lis Alban

**Affiliations:** 1 Faculty of Veterinary Medicine, University of Lisbon, Avenida da Universidade Técnica, 1300-477 Lisbon, Portugal; 2 Department of Large Animal Sciences, Section for Animal Welfare and Disease Control, University of Copenhagen, Grønnegårdsvej 8, Frederiksberg C, DK-1870 Denmark; 3 Danish Agriculture & Food Council, Axeltorv 3, Copenhagen V, DK-1609 Denmark

**Keywords:** Antimicrobial consumption, Alternatives, Vaccination, Pigs, VetStat, Denmark

## Abstract

**Background:**

There is growing concern about development of antimicrobial resistance due to use of antimicrobials (AMs) in livestock production. Identifying efficient alternatives, including vaccination, is a priority. The objective of this study was to compare the herd-level amount of AMs prescribed for weaner pigs, between Danish sow herds using varying combinations of vaccines against Porcine Circovirus Type 2 (PCV2), *Mycoplasma hyopneumoniae* (MYC) and *Lawsonia intracellularis* (LAW). It was hypothesised that herds purchasing vaccines, use these to prevent disease, and hence reduce their AM consumption, compared to herds purchasing fewer or no vaccines against these pathogens.

Data summarised over year 2013 were obtained from the Danish Central Husbandry Register and the Danish VetStat database, in which prescriptions of medication are recorded. All one-site indoor pig herds with >50 sows and >200 weaners were selected. AMs prescribed for weaners was measured in animal daily doses (ADD) and divided according to three indication groups (gastro-intestinal, respiratory indication or total use). The analysis was based on three multivariable linear regression models of the herd-level ADD for each indication group. The eight vaccination combinations (2x2x2) were included as one explanatory variable, and herd size, measured as the number of weaner pen places was included in the models as a potential confounder.

**Results:**

Out of the 1513 herds in the study, 1415 had AMs prescribed for gastro-intestinal disorders, and 836 for respiratory disorders. PCV2 vaccines were purchased in 880 herds, MYC vaccines in 787 and LAW vaccines in 115 herds. Herds purchasing PCV2 and MYC vaccines had significantly more AMs prescribed than herds not purchasing vaccines or only purchasing LAW vaccines.

**Conclusion:**

In the present study, using register data covering 1 year, we found an association between use of vaccination and increased amount of AMs prescribed for weaners. This does not exclude that the vaccines work, just that we were unable to detect this. The findings might be explained by some herds experiencing clinical problems associated with MYC or PCV2 despite use of vaccination. In other herds, it might reflect that vaccines applied to weaners are used for disease prevention in finishers rather than in the weaners. Information about vaccination protocols and herd health status was not available at the time of the study. Hence, further studies are required to investigate causality of the associations between use of AMs, vaccination practices and other confounding on-farm factors.

## Background

In Denmark, there has been political and public focus on the use of antimicrobials (AMs) in livestock and the risk of development of AM resistance since the 1990s. Focus is in particularly on the Danish pig industry, because it is the largest livestock industry in Denmark; around 28 million pigs are produced annually, and around 10 million of these are exported as weaners [1]. As a result, a series of events emerged in and around the pig industry: (a) in 1995, the veterinary profit from sales of AMs was officially limited to 5-10 % [2]; (b) in 1998, an industry initiative leading to the phasing out of growth promoters was introduced for finishing pigs, and this was expanded to weaners in 1999 (effective from January 1, 2000) [3] (c) increased surveillance and regulation of veterinary practice and prescriptions was undertaken [4], as well as (d) recommendations and guidelines for prudent use of AMs were developed. Moreover, in 2010, an industry-driven ban was implemented to stop the use of cephalosporins in pigs produced in Denmark [1].

To support the Danish policy, data regarding medical consumption for production animals are collected in a national database called VetStat established mid-2000 [5]. VetStat collects prescription records from pharmacies, feed mills and veterinary practitioners [5]. A prescription record includes information about the type, concentration and amount of AMs, the treatment indication, the age group, the individual herd number, the date of issue, the name of the veterinarian prescribing, and the name of the producer [5].

Between 2008 and the first half of 2010, the AM consumption in pigs increased, leading to a public debate [1]. Consequently, in 2010 the Danish veterinary authorities adopted the “Yellow Card” initiative, a scheme that sets permit limits to AM use in swine herds [1]. Until June 2013, the Yellow Card permit limit for weaners was 28 ADD per 100 weaners per day. Thereafter, the permit limit was reduced to 25 ADD per 100 animal days [1] and www.foedevarestyrelsen.dk. By November 2014, the permit limits were further reduced to 22.9 ADD per 100 weaners per day (Please see www.foedevarestyrelsen.dk for further updates).

The introduction of the Yellow Card scheme reflected the political pressure that is forcing Danish pig producers to reduce the usage of AMs on their farms. Therefore, efficient alternatives to routinely applied AMs have become crucial.

Vaccines are being considered a potential tool to decrease the burden of animal diseases and also to reduce the need for AMs with therapeutic purposes [6, 7].

According to data from VetStat, the three most commonly used vaccines in Danish pig production is against *Mycoplasma hyopneumoniae* (MYC) (36 %), Porcine Circovirus Type 2 (PCV2) (26 %), and *Actinobacillus pleuropneumoniae* (APP) (8 %), whereas only 3 % of the vaccine doses were prescribed for *Lawsonia intracellularis* (LAW) and only 2 % for Porcine Reproductive and Respiratory Syndrome (PRRS). These endemic disease agents are representing common production-related diseases in weaners and finishers in modern pig production. We decided to focus on the effect of MYC, PCV2 and LAW. We included MYC and PCV2 because they are the most commonly used vaccines, and we included LAW to have a vaccine with an effect on gastro-intestinal lesions. We excluded PRRS for two reasons: 1) low use and 2) apparently, in Denmark PRRS vaccines are used more commonly in breeding animals than in weaners.

PCV2 has a causal role in a large number of clinical syndromes, which are collectively named as Porcine Circovirus Diseases (PCVDs) and is highly prevalent worldwide [8]. The most economically significant condition within PCVDs is post-weaning multi-systemic wasting syndrome (PMWS) [8]. However, PCV2 can also play a role in the occurrence of reproductive failure, enteritis, porcine dermatitis and nephropathy syndrome and proliferative necrotizing pneumonia [9]. Furthermore, when there is an interaction between bacterial and viral agents, the syndrome is called porcine respiratory disease complex (PRDC) [10].

MYC is the primary agent responsible for swine enzootic pneumonia, which is a chronic respiratory disease that causes significant economic losses worldwide and is highly prevalent in most areas of pig production (present in between 38 to 100 % of the pig farms world-wide) [9]. MYC predisposes the infected animals to secondary infections, which can increase the severity of the disease for example seen as PRDC [9].

LAW is the causative bacterium of proliferative enteropathy, and it is a common high prevalence intestinal infection worldwide including Denmark [11]. This has a direct impact on pig production and herd economics as it affects growing pig performance due to the decrease in growth rates and feed conversion in some herds [12, 13].

PCV2, MYC and LAW can be controlled and prevented by different interventions. In Denmark, these include all in/all out production, multisite production, increased hygiene, antibiotic medication and use of vaccination. In Denmark, these vaccines have been used as an alternative to antibiotic medication and to increase productivity in weaners and finishers. The use of these vaccines has increased substantially since 2010. This was observed in particular right after the introduction of the Yellow Card, e.g. the use of vaccines against PCV2 infections increased by 31 % [1].

To explore the potential of using vaccination as an alternative to AMs, the present study was carried out using data from VetStat and the Danish Central Husbandry Register (CHR) from all one-site pig herds with >50 sows and >200 weaners in the year 2013. The objective was to compare the total amount of AMs prescribed for weaning pigs between Danish sow herds using varying combinations of vaccines against PCV2, MYC and LAW that year. It was assumed that year 2013 represented a steady-state in the use of vaccines and AMs. Hence, bias caused by confounding factors related to dynamics in overall health and production conditions including changes in legislation and market forces could be avoided. It was also assumed that the AMs and vaccines prescribed were used in the herd.

## Results

### Basic statistics

The median number of sows in the 1513 herds was 435 and the maximum was 3100 sows. For weaners, the median number of pen places was 1500 whereas the maximum was 21,000. Around half (52 %) of the herds also had production of finishing pigs on the same premises.

Out of the 1513 herds selected for the study, 1415 herds had AMs prescribed for gastrointestinal disorders in weaners, and 836 herds had AMs prescribed for respiratory disorders, corresponding to 94 and 55 % of the herds, respectively. There were no herds with no AMs prescribed.

With respect to total AM consumption (AC-TOTAL), the median use was 10.0 ADD per 100 weaners per day (Min: 0.004; Max: 79.33). The herd-level distribution of the total AM consumption was not normal but positively skewed with 58 herds corresponding to 4 % having AM consumption above 28 ADD per 100 weaners per day, which was the initial Yellow Card threshold.

The main part (67 %) of the AM consumption for weaners was prescribed for gastro-intestinal disorders. The overall median was 6.9 ADD per 100 weaners per day (Min: 0.02; Max: 67.16). The herd-level distribution of AM consumption with gastro-intestinal indication (AC-GI) was not normal, but positively skewed with 20 herds having AM consumption above 28 ADD per 100 weaners per day.

The median AM consumption with respiratory indication (AC-RESP) was 2.5 ADD per 100 weaners per day (Min: 0.01; Max: 56.20). The herd-level distribution of AM consumption with respiratory indication was not normal; and 17 of the herds had AM consumption above 28 ADD per 100 weaners per day.

Concerning the vaccines, 58 % (*n* = 880) of the herds purchased PCV2 vaccines in 2013. MYC vaccination was purchased in 52 % (*n* = 787) of the herds, and LAW vaccination was the least used vaccine, with just 8 % (*n* = 115) of the herds having at least one registered purchase of Enterisol®Ileitis. A total of 380 herds did not have any of these three vaccines prescribed in 2013 (Table 1).

**Table 1:** Final multivariable model* of the associations between the use of vaccines and total consumption of antimicrobials (AC-TOTAL) measured as Animal Daily Doses (ADD) per 100 weaners per day in 1513 Danish sow herds after controlling for production size, 2013. Group 0 (no vaccination) and small herd size were used as reference classes

Variables and classes	AC-TOTAL (ADD/100 weaners/day)			Converted AC-TOTAL (ADD/100 weaners/day)		
	Estimate of square root-transformed outcome	Standard error	*P*-value	Mean estimate	Lower 95 % CI	Upper 95 % CI
Intercept	2.573	0.0732	<0.0001	6.6	5.9	7.4
Combinations of vaccines			<0.0001			
Group 0: PCV2 = 0 & MYC = 0 & LAW = 0 (*n* = 380)^a^	0.000	n.a.		6.6	n.a.	n.a.
Group 1: PCV2 = 1 & MYC = 0 & LAW = 0 (*n* = 290)^b,d^	0.324	0.096		8.4	7.3	9.5
Group 2: PCV2 = 0 & MYC = 1 & LAW = 0 (*n* = 221)^b,c,d^	0.433	0.103		9.0	7.9	10.3
Group 3: PCV2 = 0 & MYC = 0 & LAW = 1 (*n* = 21)^a,b,c,d^	0.192	0.274		7.6	5.0	10.9
Group 4: PCV2 = 1 & MYC = 1 & LAW = 0 (*n* = 507)^c,d^	0.635	0.084		10.3	9.3	11.4
Group 5: PCV2 = 1 & MYC = 0 & LAW = 1 *n* = 35)^a,d^	0.083	0.216		7.1	5.0	9.5
Group 6: PCV2 = 0 & MYC = 1 & LAW = 1 (*n* = 11)^a,b,c,d^	−0.118	0.374		6.0	3.0	10.2
Group 7: PCV2 = 1 & MYC = 1 & LAW = 1 (*n* = 48)^a,b,c,d^	0.202	0.188		7.7	5.8	9.9
Herd size (number of weaner pen places)			<0.0001			
Small (*n* = 528)^a^	0.000	n.a.		6.6	n.a.	n.a.
Medium (*n* = 607)^b^	0.264	0.073		8.0	7.3	8.9
Large (*n* = 378)^c^	0.459	0.083		9.2	8.2	10.2

*n.a.* not applicable, *PCV2* Porcine Circovirus Type 2, *MYC Mycoplasma hyopneumoniae*, *LAW Lawsonia intracellularis*

^a, b, c, d^ – different letters indicate variable classes with significantly different parameter estimates of antimicrobial consumption according to an F-test

*Model statistics: *R* ^*2*^ = 0.07, *F* = 12.2, *P* < 0.001

### Results of multivariable analyses

With respect to total AM consumption (Table 1), the variable herd size and the variable representing vaccine use were both statistically significant (*P* < 0.0001). Some degree of confounding between herd size and the vaccine use variable was observed but only for the parameter describing use of PCV2 and LAW (*n* = 35 herds). The interaction between herd size and vaccine use was non-significant (*P* = 0.48). A model with vaccine use and herd size only explained 7 % of the variance in the total AM consumption (*R* ^*2*^ = 0.07, *F* = 12.2, *P* < 0.001). The highest use was observed in Group 4 (10.3 ADD/100 weaners/day), representing use of MYC and PCV2 vaccination, whereas the lowest consumption was observed in Group 6 (6.0 ADD/100 weaners/day), representing use of MYC and LAW vaccination. Group 1, 2, and 4, representing three different combinations of use of MYC and PCV2 vaccination, were all associated with a statistically higher AM consumption than the use of no vaccine at all (group 0); between 1.8 and 3.7 higher ADD per 100 weaners per day compared with group 0. The remaining vaccine combination groups were not associated with statistically different levels of AM consumption compared to the group not using any of the three vaccines (*P* > 0.05). Smaller herds had a significantly lower total AM consumption than medium-sized herds (6.6 versus 8.0 ADD/100 weaners/day), which again had a significantly lower consumption than large herds where the mean AM consumption was 9.2 ADD/100 weaners/day (Table 1).

Regarding AM consumption for gastro-intestinal indication (Table 2), only herd size was statistically significant (*P* = 0.02). Some degree of confounding between herd size and the vaccine use variable was observed but only for the parameter related to the group using LAW vaccine alone (*n* = 21 herds). The variable describing the vaccine use had a *P*-value of 0.2, and the interaction term between herd size and vaccine use had a *P*-value of 0.37. A model including vaccine use and production size only explained 0.6 % of the variance in the AM consumption (*R* ^*2*^ = 0.006, *F* = 2.0, *P* = 0.03) (Table 2). Large herds had significantly higher AM consumption than small herds (6.8 versus 5.7 ADD/100 weaners/day), whereas the AM consumption in medium-sized herds were in between. A detailed look into Table 2 shows that the lowest AM consumption was observed in Group 0, 2, 3 and 6 (5.7–6.0 ADD/100 weaners/day) and the highest use in Group 5 (7.0 ADD/100 weaners/day).

**Table 2:** Final multivariable model* of the associations between use of vaccines and consumption of antimicrobials with gastro-intestinal indications (AC-GI) in 1415 Danish sow herds after controlling for production size, 2013. Group 0 (no vaccination) and small herd size were used as reference classes

Variables and classes	AC-GI (ADD/100 weaners/day)			Converted AC-GI (ADD/100 weaners/day)		
	Estimate of square root-transformed outcome	Standard error	*P*-value	Mean estimate	Lower 95 % CI	Upper 95 % CI
Intercept	2.384	0.076	<0.0001	5.7	5.0	6.4
Combinations of vaccines			0.2			
Group 0: PCV2 = 0 & MYC = 0 & LAW = 0 (*n* = 351)	0.000	n.a.		5.7	n.a.	n.a.
Group 1: PCV2 = 1 & MYC = 0 & LAW = 0 (*n* = 277)	0.145	0.097		6.4	5.5	7.4
Group 2: PCV2 = 0 & MYC = 1 & LAW = 0 (*n* = 204)	−0.004	0.106		5.7	4.7	6.7
Group 3: PCV2 = 0 & MYC = 0 & LAW = 1 (*n* = 21)	0.021	0.270		5.8	3.5	8.6
Group 4: PCV2 = 1 & MYC = 1 & LAW = 0 (*n* = 476)	0.183	0.085		6.6	5.8	7.5
Group 5: PCV2 = 1 & MYC = 0 & LAW = 1 (*n* = 32)	0.258	0.222		7.0	4.9	9.5
Group 6: PCV2 = 0 & MYC = 1 & LAW = 1 (*n* = 9)	0.058	0.405		6.0	2.7	10.5
Group 7: PCV2 = 1 & MYC = 1 & LAW = 1 (*n* = 45)	−0.026	0.190		5.6	3.9	7.5
Herd size (number of weaners pen places)			0.02			
Small (*n* = 468)^a^	0.000	n.a.		5.7	n.a.	n.a.
Medium (*n* = 585)^a,b^	0.151	0.075		6.4	5.7	7.2
Large (*n* = 362)^b^	0.227	0.085		6.8	6.0	7.7

*n.a.* not applicable, *PCV2* Porcine Circovirus Type 2, *MYC Mycoplasma hyopneumoniae*, *LAW Lawsonia intracellularis*

^a, b^ – different letters indicate variable classes with significantly different parameter estimates of antimicrobial consumption according to an F-test

*Model statistics: *R* ^*2*^ = 0.006, *F* = 2.0, *P* = 0.03

In relation to the AC with respiratory indication (Table 3), herd size was non-significant (*P* = 0.06) but some confounding between herd size and vaccine use was observed for two of the parameter estimates of the vaccine variable (use of LAW vaccine alone: 10 herds; use of MYC and LAW vaccines: 6 herds). The interaction term between herd size and vaccine use was non-significant (*P* = 0.80), whereas the variable describing vaccine use was statistical significant (*P* < 0.0001), but it only explained 4 % of the variance in the AM consumption (*R* ^*2*^ = 0.04, *F* = 4.8, *P* = 0.001). The lowest AM consumption was observed in Group 5, representing the use of PCV2 and LAW vaccination (0.6 ADD/100 weaners/day). However, this was not significantly different from Group 0, statistically speaking (1.6 ADD/100 weaners/day). Group 2 and 4 representing use of MYC vaccination with and without concurrent use of PCV2 vaccination were associated with a statistically higher AM consumption than the use of no vaccines (Group 0) – 3.1 and 3.2 versus 1.6 ADD/100 weaners/day. The changes in AM consumption associated with the rest of the combinations were not statistically significant (*P* > 0.05).

**Table 3:** Final multivariable model* of the associations between use of vaccines and consumption of antimicrobials with respiratory indications (AC-RESP) in 836 Danish sow herds after controlling for production size, 2013. Group 0 (no vaccination) and small herd size were used as reference classes

Variables and classes	AC-RESP (ADD/100 weaners/day)			Converted AC-RESP (ADD/100 weaners/day)		
	Estimate of log-transformed outcome	Standard error	*P*-value	Mean estimate	Lower 95 % CI	Upper 95 % CI
Intercept	0.497	0.145	<0.0001	1.6	1.2	2.2
Combinations of vaccines			<0.0001			
Group 0: PCV2 = 0 & MYC = 0 & LAW = 0 (*n* = 155)^a,c^	0.000	n.a.		1.6	n.a.	n.a.
Group 1: PCV2 = 1 & MYC = 0 & LAW = 0 (*n* = 136)^a^	0.404	0.177		2.5	1.7	3.5
Group 2: PCV2 = 0 & MYC = 1 & LAW = 0 (*n* = 134)^b^	0.648	0.177		3.1	2.2	4.4
Group 3: PCV2 = 0 & MYC = 0 & LAW = 1 (*n* = 10)^a,c^	0.160	0.490		1.9	0.7	5.0
Group 4: PCV2 = 1 & MYC = 1 & LAW = 0 (*n* = 349)^b^	0.672	0.146		3.2	2.4	4.3
Group 5: PCV2 = 1 & MYC = 0 & LAW = 1 (*n* = 13)^c^	−0.994	0.434		0.6	0.3	1.4
Group 6: PCV2 = 0 & MYC = 1 & LAW = 1 (*n* = 6)^a,b,c^	−0.166	0.626		1.4	0.4	4.7
Group 7: PCV2 = 1 & MYC = 1 & LAW = 1 (*n* = 33)^a,b,c^	0.220	0.289		2.0	1.2	3.6
Herd size (number of weaners pen places)			0.06			
Small (*n* = 235)	0.000	n.a.		1.6	n.a.	n.a.
Medium (*n* = 331)	−0.261	0.129		1.3	1.0	1.6
Large (*n* = 270)	−0.295	0.136		1.2	0.9	1.6

*n.a.* not applicable, *PCV2* Porcine Circovirus Type 2, *MYC Mycoplasma hyopneumoniae*, *LAW Lawsonia intracellularis*

^a, b, c^ – different letters indicate variable classes with significantly different parameter estimates of antimicrobial consumption according to an F-test

*Model statistics: *R* ^*2*^ = 0.04, *F* = 4.8, *P* = 0.001

## Discussion

### General discussion

Almost all herds (96 %) had a total AM consumption lower than 28 ADD per 100 weaners per day This implies that the majority of the producers were able to raise weaners while fulfilling the requirements regarding AM consumption set by the Danish veterinary authorities.

The statistical analyses showed that in general the pig herds using vaccines against MYC, PCV2 and LAW had a higher – and not as hypothesised a lower AM consumption – in the weaning stage compared to not using vaccination at all. This is in line with results presented by Potsma et al. (2016) [14]. This may be explained by some herds experiencing clinical problems associated in particular with MYC or PCV2 despite use of vaccination. The herds not applying vaccination are probably herds with a high health status, where these three infections are not present – or at least not causing clinical problems. In other herds, the lack of impact of vaccination on the AM consumption in weaners might reflect that vaccines are used for disease prevention in finishers rather than in the weaners as pointed out by Raidt et al. [15]. Alternatively, other infections may be present which we could not adjust for in the statistical analyses. Moreover, in Denmark much has already been done to lower the antimicrobial consumption; latest with the Yellow Card scheme setting limitations to the consumption in an age group such as the weaners as described above. Finally, the lack of effect of the vaccine combinations on the AM with gastro-intestinal indication may be explained by the fact that only the LAW vaccine has a direct impact on gastro-intestinal infections.

### Effect of PCV2 vaccination

Control of PCV2-related diseases has traditionally been based on preventive measures such as: (1) improved management practices in order to control risks or triggering factors, (2) control of concurrent infections and (3) changes of the boar genetic background [8]. Currently, disease control is mainly based on vaccination, which has been shown to be very effective in reducing viraemia, improving production parameters (e.g. reducing mortality and increasing average daily weight gain) and the probability of co-infection by other pathogens [16–18].

In the present study, herds using PCV2 vaccination had a statistically significantly higher total AM consumption and AM consumption with respiratory indication compared to herds not using the vaccines – except from the case when LAW vaccination was applied as well (Table 1). This is contrary to results presented by Raidt et al. [15] who followed the consumption of antimicrobials in 65 Austrian swine herds after the first licensing of the PCV2 in Austria. The Danish results can probably be explained by reverse causality hereby pointing to PCV2’s presence in the herds and its ability to cause disease. Moreover, as explained above, there may be an effect of vaccination on the finishers as shown by [15].

As described above, PCV2 has been linked with various clinical syndromes in different organs. This might justify the application of a major amount of AMs [19, 20]. PCV2 can be considered a necessary but not sufficient factor to develop clinical disease [17]. Therefore, farmers and practitioners could very well have decided to routinely use PCV2 vaccination in these herds to avoid this predisposing factor and consequently, the occurrence of PCVDs.

### Effect of MYC vaccination

Use of vaccination against MYC has been shown to be associated with reduced clinical signs, fewer treatment costs and with increased average daily weight gain [21, 22]. Vaccination is therefore considered the most adequate measure for controlling MYC infection in practice [23]. Vaccination is performed in piglets, weaners and to a smaller extent in grower-finishing pigs. Vaccination of piglets during lactation is the most common. Vaccination of replacement gilts and sows is also performed in some herds.

According to the results of the multivariable analysis, on average herds using MYC vaccination had a higher total AM consumption and AM consumption with respiratory indication than herds not using the vaccines. This finding may be explained by reverse causality: herds using the vaccines were likely infected with this pathogen – some with clinical problems. Subsequently, these herds were using AMs with respiratory indication to control MYC and, simultaneously using the vaccines to avoid the predisposition of the infected animals to secondary invaders, especially other pulmonary pathogens [9]. Other herds neither vaccinated nor treated, presumably because they were not infected with MYC.

### Effect of LAW vaccination

In 2013, only one vaccine was available in Denmark against proliferative enteropathy caused by LAW. It is used for piglets in the last part of lactation or (more commonly) in the first week post weaning. The positive effect of the LAW vaccine has been shown by Bak & Rathkjen, who undertook a study in a Danish Specific Pathogen Free herd [12]. Here, proliferative enteropathy was prevented by use of the vaccination and improved growth rate and a reduction in the use of AMs was observed after initiation of vaccination. However, the positive effect may be more limited in non-SPF herds due to presence of other infections. But Thaker & Bilkei (2006) also concluded that vaccination reduced LAW-associated losses as well as improved health and the immune state of pigs in highly infected herd [24].

As LAW vaccination is used to prevent proliferative enteropathy [12], it would be expected that herds using the vaccination would have been associated with a lower AM consumption with gastrointestinal indication – or lower total AM consumption. However, there was no statistically significant effect of the vaccine on AM consumption in the weaner section (neither the total AM consumption, nor the consumption with a gastro-intestinal indication or respiratory indication). There were some indications that use of LAW vaccination was associated with a lower AM consumption with respiratory indication – but the findings were not statistical significant (Table 3). This may be because LAW vaccine has no effect on respiratory disease. However, to some extent AMs are being used for other disease categories than those officially prescribed for. Certain AMs, as for example some doxycyclines, are not officially registered for treating infections with gastro-intestinal indication in Denmark, although they are known to be effective in everyday practice. This may result in recordings indicating that these AM were used for respiratory indication although the aim was to treat gastro-intestinal infections.

### Considerations, limitations and further work for the study

We assumed that the AM and vaccines prescribed in a herd were also used in the same herd. For some countries there may be a difference between prescription and use. However, for Denmark it is the general belief that VetStat data (consisting of prescription data) approximate AM use closely over a longer period of time. One reason is that it is the only legal way to get AM for livestock in Denmark. This was underpinned by a recent report from the Danish veterinary authorities stating that there are no indications of a systematic illegal import of AMs [25].

On average, 36 % of the total amount of AMs used for the production of a pig until slaughter is used during the weaning period [26] although weaners only cover the production from 7 to 30 kg, which corresponds to around 4 weeks. This reflects that the treatment incidence is much higher in weaners compared with finishers and sows [24]. This is one of the reasons why the present study focused on the AM consumption for weaners. However, it would also be of interest to study the effect of vaccination in the sow herd on the consumption of AM in the finishing section. On the other hand, according to Potsma et al. [14] a higher AM consumption in sows tended to be associated with higher AM consumption from birth until slaughter, and that it was positively associated with the number of pathogens vaccinated against.

The multivariable models presented in Tables 1, 2 and 3 only explained a limited amount of the variation in the data. This shows that many other factors – apart from vaccination – determine the need for AM in a given pig herd. Information about such factors was not available at the time of the study e.g. regarding (1) vaccination protocols applied including age of pigs at time of vaccination (2) initiation and duration of vaccination (3) use of other vaccines that were part of the general vaccination program (4) herd health status including presence of other infections, (5) internal and external biosecurity (6) management practices (7) turnover of animals in each herd (8) export of live animals (where vaccination may be required by the customer). If this information had been available, we would probably have been capable of explaining a larger degree of the variation in the data.

In this study, we focused on MYC, PCV2 and LAW. One reason for only including these three vaccines in the analysis was to avoid that the number of herds representing different combinations of vaccine use would become very low, because this would result in unstable parameter estimates. We did not take into account PRRS vaccination. Although this vaccine only represents 2 % of the vaccines prescribed for pigs in Denmark, it could have been a confounder in the analyses, and it would therefore be of interest to include this in a subsequent analysis. Similarly, it would have been of interest to include also vaccines with an effect on APP. However, this was not done in this analysis due to the limited use (8 % of the prescribed vaccine doses prescribed to Danish swine). It is also possible that vaccination of breeding animals for *E. coli* to prevent *E. coli* associated neonatal diarrhea in piglets, could result in higher piglet health at weaning and therefore also affect the medical consumption post weaning. But the contrary is also possible; that herds applying the vaccine do this because they have clinical problems with neonatal diarrhea infections requiring antimicrobial treatment despite the use of vaccination.

This study was a first basic approach to using register data and a cross-sectional design to describe the possible association between vaccinations and AM use in pig herds in Denmark. The vaccine data from VetStat have to the authors’ knowledge not been used in analysis of AM use before. It is not possible to elucidate the directions of these associations. A longitudinal study will enable a better understanding of cause and effect and be able to take into account other factors than just vaccination.

More information is needed to assess to which extent vaccinations – and other preventive measures - can in fact reduce the need for the use of AMs. The feasibility of using vaccination as a an alternative to AMs will depend on proper disease diagnostics, the costs of vaccines compared to AM, effectiveness and ease of use [6]. If in the future we get an affirmative answer to this question and farmers can see return on their investment, improvement of pig health and productivity will occur through a wider application of routine vaccination instead of routine AM treatments.

## Conclusions

In general, the sow herds applying MYC and PCV2 vaccination had more AMs prescribed for weaners compared to sow herds not using the three studied vaccines – probably as a result of existing health problems in the herds prior to and/or during the use of vaccination. For LAW vaccine there was a trend towards lower or equal amount of prescribed AMs compared to herds not purchasing any of the three vaccines. These results suggest that vaccination alone does not necessarily come along with a low use of AMs, despite being an asset in many regards.

Each herd has its own challenges and several issues need to be taken into account when it concerns alternatives to AM consumption. Further studies need to be carried out to take into consideration other factors regarding prevention of disease, which are of extreme importance, such as biosecurity and management practices within the herds.

## Methods

### Data

The data used in this study were obtained from two sources. The first was the VetStat database, which contains information about all prescriptions of AM vaccines destined for livestock. VetStat quantifies the AM prescriptions in food animal using the unit animal daily dose (ADD), which is defined as the average maintenance dose per kg live animal for the main indication of an AM in a specified species [27]. This measure takes into account the different potency of the various AM classes. To correct for the large variation in the weight of weaners, the official standard estimate of 15 kg was applied [27, 28].

The second source of data was the Danish CHR register, which contains information about location and size of all livestock herds. All pig herds were selected for the study, if they fulfilled the following inclusion criteria: a) one-site indoor herds b) herds with more than 50 sows and c) herds with more than 200 weaners. In total 1518 herds met these three criteria, and data regarding the prescription of AMs for weaners as well as the number of vaccine doses purchased in 2013 were obtained for each of these herds from VetStat.

Five herds had erroneous (e.g. negative values of recorded AM or vaccine use) or missing data records. All cases were most likely caused by an error in VetStat. As all the herds in the dataset were anonymised, it was not possible to assess the reasons for these data errors. Consequently, these five herds were excluded from the analysis.

For each of the remaining 1513 herds, a variable was created to estimate the average AM consumption per 100 weaners per day. This variable was calculated using the total administered ADD in weaners in year 2013, dividing it by the standard weight of weaners used by VetStat (15 kg). After this first calculation, to have the number of ADD for each weaner, this total was divided by the number weaners (pen places) in each herd. Finally, this total was divided by 3.65, to show the final values in ADD per 100 animals per day – which corresponds to the unit used by VetStat to impose the official AM consumption limits.

Taking into account that weaners may receive more than one type of vaccine, two-way combinations between vaccination groups were constructed to assess the possible association between different vaccinations and AM consumption. Moreover, for the final models, one variable with eight levels was constructed to take into consideration all combinations of use of the three different vaccines within the herds.

### Data analyses

**Fig. 1:**
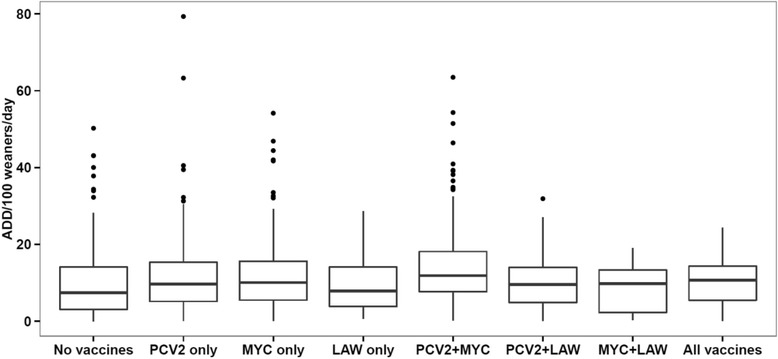
Total use of antimicrobials – measured as Animal Daily Doses (ADD) per 100 weaners per day – in 1513 Danish sow herds, divided according to the combined use of vaccination against PCV2, *Mycoplasma hyopneumoniae* (MYC), and *Lawsonia intracellularis* (LAW), 2013

**Fig. 2:**
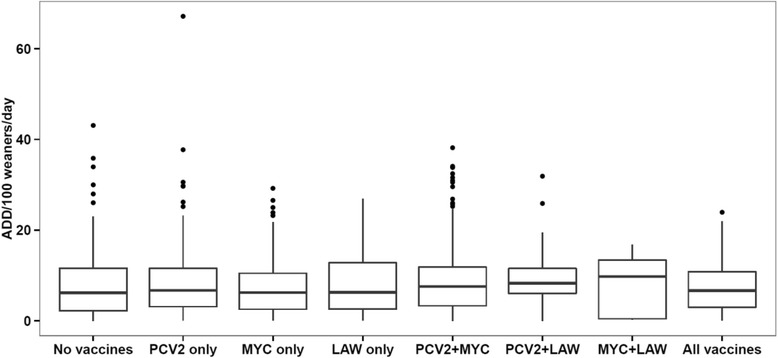
Use of antimicrobials with gastro-intestinal indication – measured as Animal Daily Doses (ADD) per 100 weaners per day – in 1415 Danish sow herds, divided according to the combined use of vaccination against PCV2, *Mycoplasma hyopneumoniae* (MYC), and *Lawsonia intracellularis* (LAW), 2013

**Fig. 3:**
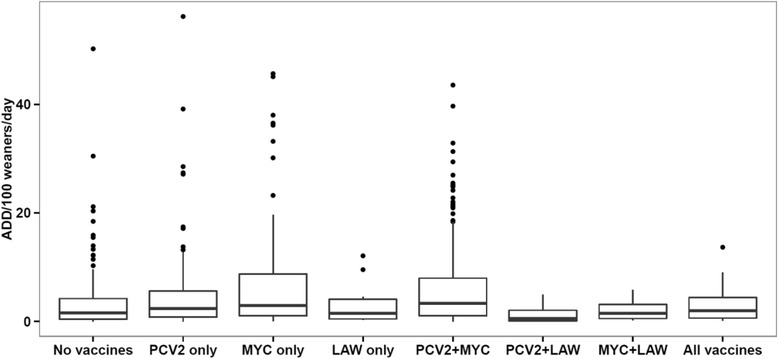
Use of antimicrobials with respiratory indication – measured as Animal Daily Doses (ADD) per 100 weaners per day – in 836 Danish sow herds, divided according to the combined use of vaccination against PCV2, *Mycoplasma hyopneumoniae* (MYC), and *Lawsonia intracellularis* (LAW), 2013

All data analyses were carried out in R (version 3.1.2 of 2014 – The R Foundation for Statistical Computing). Univariable analyses were performed for the total AM consumption (AC-TOTAL), AM consumption with gastrointestinal indication (AC-GI) and AM consumption with respiratory indication (AC-RESP) Figs. 1, 2 and 3). Herd size – measured as number of weaner pen places – was divided into three classes: small (<7500), medium (7500–14,999), and large (≥15,000). AC-TOTAL and AC-GI were square root transformed whereas AC-RESP was log-transformed to normalize the distributions. Regarding vaccination: First we went through a pre-analysis step involving the creation of a vaccination coverage index based on the prescriptions of vaccines in 2013. However, it turned out not very useful, so we decided to assign herds as vaccinated if the respective type(s) of vaccine(s) had been prescribed during 2013 irrespective of the number of doses prescribed.

Initially, a t-test was conducted for each of the three types of vaccination comparing the AM consumption for herds which used the vaccine with herds which did not use the vaccine.

For each of the two-way combinations, a t-test and a one-way ANOVA were conducted. Following the one-way ANOVA, a post hoc comparison – using the TUKEY HSD test – was performed to assess the statistical difference between the individual combinations (Data not shown).

Finally, multivariable analyses were conducted for (1) AC-TOTAL, (2) AC-GI and (3) AC-RESP as three separate outputs. The variables herd size and use of vaccine (divided into the eight different combinations of use of the three vaccines) represented the explanatory variables. It was tested whether herd size was significantly associated with the response and whether it acted as a confounder by being associated with the vaccine use. Moreover, a test was made for presence of interaction between herd size and vaccine. A *P*-value < 0.05 was used as threshold for statistical significance. As part of model validation, residuals were inspected for normal distribution.

## Abbreviations

AC-GI, antimicrobial consumption with gastro-intestinal indication; AC-RESP, antimicrobial consumption with respiratory indication; AC-TOTAL, total antimicrobial consumption; ADD, animal daily doses; AM, antimicrobial; CHR, central husbandry register; LAW, *Lawsonia intracellularis*; MYC, *Mycoplasma hyopneumoniae*; PCV2, porcine circovirus type 2; PCVD, porcine circovirus diseases; PMWS, post-weaning multi-systemic wasting syndrome; PRRS, porcine reproductive and respiratory syndrom
